# Jawbone Cavitations: Current Understanding and Conceptual Introduction of Covered Socket Residuum (CSR)

**DOI:** 10.3390/bioengineering13010106

**Published:** 2026-01-16

**Authors:** Shahram Ghanaati, Anja Heselich, Johann Lechner, Robert Sader, Jerry E. Bouquot, Sarah Al-Maawi

**Affiliations:** 1 Department of Oral, Cranio-Maxillofacial and Facial Plastic Surgery, Goethe University Frankfurt, 60590 Frankfurt am Main, Germany; 2 FORM-Lab, Frankfurt Orofacial Regenerative Medicine Laboratory, Goethe University Frankfurt, 60590 Frankfurt am Main, Germany; 3 Clinic for Integrative Dentistry, 81547 Munich, Germany; 4 Department of Oral & Maxillofacial Surgery, West Virginia University, Morgantown, WV 26506-9400, USA

**Keywords:** Covered Socket Residuum (CSR), cavitation, post-extraction healing, Guided Open Wound Healing (GOWH), Platelet-Rich Fibrin (PRF), bone regeneration, critical size defect, Neuralgia-Inducing Cavitational Osteonecrosis (NICO), Fatty Degenerative Osteolysis of the Jawbone (FDOJ)

## Abstract

Jawbone cavitations have been described for decades under various terminologies, including neuralgia-inducing cavitational osteonecrosis (NICO) and fatty degenerative osteolysis of the jawbone (FDOJ). Their biological nature and clinical relevance remain controversial. The present review aimed to summarize the current understanding of jawbone cavitations, identify relevant research gaps, and propose a unified descriptive terminology. This narrative literature review was conducted using PubMed/MEDLINE, Google Scholar, and manual searches of relevant journals. The available evidence was qualitatively synthesized. The results indicate that most published data on jawbone cavitations are derived from observational, retrospective, and cohort studies, with etiological concepts largely based on histopathological findings. Recent three-dimensional radiological analyses suggest that intraosseous non-mineralized areas frequently observed at former extraction sites may represent a physiological outcome of socket collapse and incomplete ossification rather than a pathological condition. This review introduces Covered Socket Residuum (CSR) as a radiological descriptive term and clearly distinguishes it from pathological entities such as NICO and FDOJ. Recognition of CSR is clinically relevant, particularly in dental implant planning, where unrecognized non-mineralized areas may compromise primary stability. The findings emphasize the role of three-dimensional radiological assessment for diagnosis and implant planning and discuss preventive and therapeutic strategies, including Guided Open Wound Healing (GOWH^TM^). Prospective controlled clinical studies are required to validate this concept and determine its clinical relevance.

## 1. Introduction

The formation of cavitations within the jaw bones has been described for several decades [1,2,3]. From their initial description, jawbone cavitations were reported in association with facial neuralgias, related pain syndromes, and other clinical symptoms such as hypaesthesia [1]. These observations have led to the hypothesis that jawbone cavitations may be clinically relevant in selected patients [4]. However, a causal relationship has not been conclusively established yet [5]. In addition, some authors have suggested potential associations between jawbone cavitations and systemic chronic conditions, including rheumatic [6], neuralgic [7], and chronic inflammatory diseases [8]. These reports are largely based on observational studies and retrospective analyses [8,9]. Notably, jawbone cavitations have also been identified in otherwise healthy individuals without corresponding clinical symptoms, further complicating the interpretation of their clinical significance [10].

Over time, reports and studies about jawbone cavitations have appeared in the literature under a wide range of terminologies, such as neuralgia-inducing cavitational osteonecrosis (NICO) [11,12,13] or fatty degenerative osteolysis of the jawbone (FDOJ) [8], and jawbone cavitations [14]. This heterogeneity in terminology additionally led to confusion and significantly limited the scientific reproducibility as well as the comparability of reported findings.

Furthermore, reliable identification, clinical diagnosis, and causal treatment of the underlying etiology have remained challenging. This is largely due to poorly defined clinical and radiological diagnostic criteria, the lack of clinically detectable intraoral pathology, and the absence of clearly defined characteristic radiological signs in the conventional assessments [15]. At the same time, the repeated reporting of this entity reflects sustained clinical interest and suggests that these findings may be associated with a relevant patient burden in certain clinical contexts.

In addition to the clinical challenges outlined above, jawbone cavitations have remained a controversially discussed topic in the literature [15]. Although several attempts have been made to formulate evidence-based recommendations, a scientific consensus has not yet been achieved.

The aim of the present narrative review is to summarize the current understanding of jawbone cavitations, identify relevant gaps in the existing literature to outline future research questions, and propose a unified and descriptive terminology to facilitate future research and clinical communication.

## 2. Methods

The present narrative review is based on a literature search conducted using PubMed/MEDLINE, Google Scholar, and manual searches of relevant journals. Articles considered relevant to the topic of jawbone cavitations were identified and selected for inclusion in the review. The identified literature was qualitatively synthesized and discussed within the respective sections of this manuscript.

## 3. Results

Most of the found studies were observational studies, case reports or retrospective studies. Only one relevant randomized controlled study was found. The results of the literature research are presented in the following sections.

### 3.1. Etiology of Jawbone Cavitations: Current Concepts and Theories

Jawbone cavitations represent non-mineralized intraosseous areas within the jaw bones. Over the past decades, various attempts have been made to elucidate the biological and pathophysiological mechanisms underlying these observations (Table 1).

Current concepts proposed to explain the formation of jawbone cavitations are based on heterogeneous methodological approaches and implicate multiple contributing factors, mostly resulting after tooth extractions or dental treatments adjacent the bone such as root canal treatment [3,16]. Histopathological analyses of tissue samples obtained from jawbone cavitations have reported findings such as intraosseous inflammatory changes [1,17], fatty degeneration [1], and areas suggestive of osteonecrosis [17].

In addition to local tissue analyses, some studies have examined systemic parameters and reported altered blood biomarker profiles in affected patients, including increased levels of cytokines and growth factors such as C-C motif chemokine ligand 5 (CCL5) and fibroblast growth factor-2 (FGF-2) [18]. These findings have been interpreted as potential indicators of chronic inflammatory activity or dysregulated healing processes; however, their specificity for jawbone cavitations and their causal relevance remain uncertain.

Supportive evidence has been provided by a recent molecular study analyzing gene expression patterns in clinical samples obtained from jawbone cavitations—described in that study in the context of fatty degenerative osteonecrosis of the jaw (FDOJ). Using quantitative real-time polymerase chain reaction (qRT-PCR), the authors reported a significant upregulation of inflammatory mediators, including CCL5/RANTES, vascular endothelial growth factor (VEGF), insulin-like growth factor (IGF), and κ-opioid receptor (KOR), alongside a downregulation of structural proteins such as collagen types I, II, and IV, as well as osteogenesis-associated factors [19].

Other investigations have suggested potential associations between jawbone cavitations and abnormalities of the thrombotic and fibrinolytic systems. Defects in coagulation or fibrinolysis have been proposed as possible predisposing factors for impaired intraosseous healing and the subsequent development of jawbone cavitations [20].

Furthermore, the formation of jawbone cavitations has been suggested to be associated with specific dental conditions, including chronic inflammatory processes [21], previous root canal treatments [16], and wisdom tooth extractions [22]. These factors have been discussed as potential contributors to persistent inflammatory environments that may impair normal intraosseous healing and thereby be associated with the development of jawbone cavitations.

Most of the findings described above are derived from retrospective analyses, case reports, or cohort studies and primarily address the characterization of pre-existing jawbone cavitations. Consequently, evidence directly addressing the etiology of jawbone cavitation formation remains limited.

In this context, our group recently conducted a clinical study investigated the healing of premolar extraction sockets over a six-month period using three-dimensional radiological imaging to assess socket healing and explore potential morphological pathways leading to jawbone cavitations. The results indicated that premolar sockets left to heal without additional intervention frequently exhibited areas of incomplete mineralization within the jawbone, consistent with the description of jawbone cavitations [10].

A further randomized controlled clinical study evaluated the healing patterns of third molar extraction sockets treated either with platelet-rich fibrin (PRF), an autologous blood concentrate system, alone or with a combination of bone substitute material (BSM) and PRF (BSM + PRF). Healing outcomes were assessed radiologically using three-dimensional imaging and visualization techniques. The results demonstrated that sockets treated with PRF alone frequently exhibited non-mineralized intraosseous areas, whereas sockets treated with BSM + PRF showed complete mineralization [22]. Similarly to the premolar socket study, the non-mineralized areas were primarily localized in the central or apical regions of the socket and were covered by mineralized bone in the crestal region. In this context, we referred to these findings as Covered Socket Residuum (CSR) [22].

The findings of both clinical studies with radiological investigations demonstrated that the formation of CSR may be a physiological condition following unassisted socket healing. The presence of CSR is thereby not necessarily related to disease condition, but may be affected on the long term. However, further long-term research is needed to validate these findings and outline their clinical relevance and correlation with the previously described pathophysiology of jaw cavitations.

**Table 1:** Historical Development of Concepts Regarding the Etiology of Jawbone Cavitations.

Author (Year)	Study Type	Terminology Used	Primary Method	Etiological Concept
Roberts & Person (1979) [2]	Retrospective case series	Bone cavities at previous extraction sites	Clinical observation	Facial pain phenomena associated with cavitations at former extraction sites
Ratner et al. (1979) [14]	Retrospective case series	Bone cavities	Histopathological and microbiological analysis	Chronic lymphocytic inflammation with polymicrobial flora
Bouquot (1992) [23]	Retrospective observational study	Neuralgia-inducing cavitational osteonecrosis (NICO)	Histopathological examination	Chronic intraosseous inflammation, marrow fibrosis, necrotic bone fragments
Gruppo et al. (1996) [20]	Case–control study	Neuralgia-inducing cavitational osteonecrosis (NICO)	Blood coagulation and fibrinolysis analysis	Defects in thrombotic and fibrinolytic systems
Lechner & Mayer (2010) [11]	Retrospective case series	Neuralgia-inducing cavitational osteonecrosis (NICO)	Multiplex cytokine analysis	Overexpression of RANTES and IL-1
Lechner (2014) [24]	Case–control study	Fatty degenerative osteolysis of the jawbone (FDOJ)	Correlation of radiography with inflammatory markers	Marked RANTES elevation; 2D radiography insufficient
Ghanaati et al. (2025) [10]	Retrospective case–control study	Jawbone cavitation	Three-dimensional radiological assessment (CBCT)	Cavitations as result of socket collapse after tooth loss
Ghanaati et al. (2025) [22]	Prospective randomized controlled trial	Covered Socket Residuum (CSR)	Three-dimensional radiological assessment (CBCT)	Covered non-mineralized intraosseous regions after extraction

### 3.2. Conceptual Introduction of Covered Socket Residuum (CSR)

**Figure 1:**
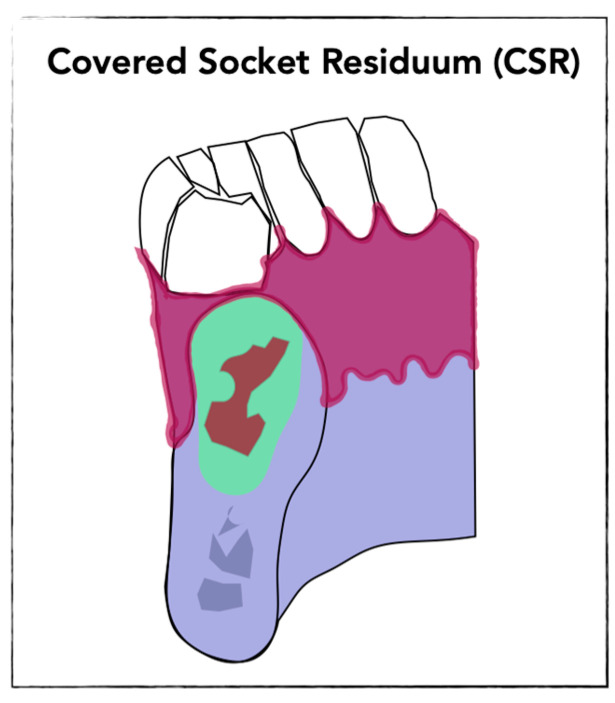
Cross-sectional depiction of the CSR showing mineralized crestal bone beneath closed mucosa and central unmineralized zone (illustrative diagram; green = newly formed bone, brown = CSR).

CSR was found in former extraction sockets, that were not stabilized by bone substitute materials. The description of CSR relies on dynamic three-dimensional radiologic investigations using innovative visualization techniques. It was firstly observed in formed premolar sockets and was described as a programmed socket collapse accompanied by the formation of cavitations within the alveolus [10]. The mechanism of socket collapse was demonstrated as a combination of two steps that physiologically take place during socket healing. First, an inward movement of the vestibular socket wall over time leading to approximation of oral and vestibular socket walls and reducing the socket defect volume from a critical-size defect to a non-critical-size defect, allowing the crestal part to mineralize. Second, new bone formation as an interaction of bone apposition along socket walls and simultaneous dimensional reduction in the alveolar ridge. Thereby, the CSR is predominantly located within the central region of the socket and covered by mineralized tissue in the crestal region [10], (Figure 1).

### 3.3. Diagnostic Approaches for CSR

**Figure 2:**
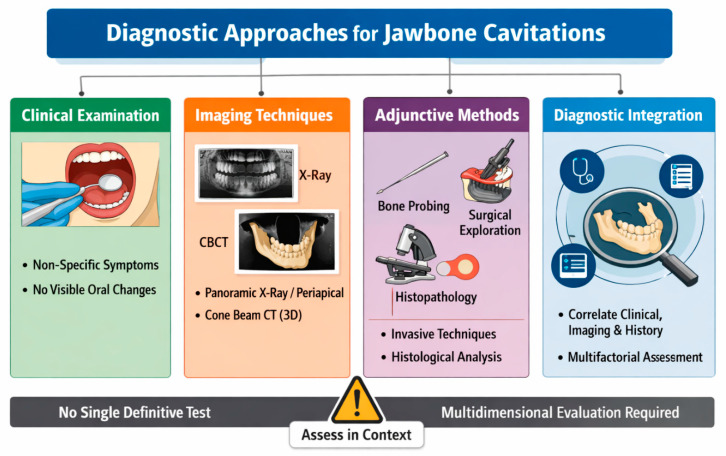
Diagnostic criteria for CSR.

Jawbone cavitations require a multidimensional diagnostic approach to enable reliable identification and correct interpretation of their clinical relevance. To date, there is no evidence-based consensus regarding standardized diagnostic criteria for jaw cavitations in general. Similarly to other jaw related diseases, diagnosis should be based on a combination of clinical findings, imaging modalities, and if needed intraoperative or histopathological assessment. The presence of jaw cavitations in terms of CSR may therefore be a physiological condition after unassisted socket healing without the need for further clarification (Figure 2).

#### 3.3.1. Clinical Assessment

Clinical examination alone is insufficient in this context, as jawbone cavitations are typically not associated with distinct intraoral pathological changes.

#### 3.3.2. Radiological Evaluation

Conventional two-dimensional X-ray is currently not sufficient for a precise detection of CSR. Advances in three-dimensional imaging, particularly cone beam computed tomography (CBCT) [10,22], have led to an increasing detection of non-mineralized areas within former extraction sockets. Non-mineralized areas within the jaw should be thereby described as CSR when following criteria are fulfilled after the application of modern visualization techniques, as preciously described [10,22]:

- Presence of non-mineralized or low-density areas within the former tooth socket area
- Non-mineralized area surrounded by mineralized bony layer in the crestal part of the jaw (figure)
- Absence of radiological criteria for other established diseases (e.g., jaw cyst and odontogeneic tumors)
- Absence of malignant criteria

When the criteria are fulfilled, CSR should be considered as a radiological diagnosis, that may present a physiological condition in asymptomatic individuals or require further histopathological clarification in selected cases.

#### 3.3.3. Histopathological Analysis

In specific cases, further diagnostic may be necessary to further outline the histopathology of CSR.

In this context, it is important to emphasize that Covered Socket Residuum (CSR) is not identical to previously described histopathological diagnoses such as fatty degenerative osteolysis of the jawbone (FDOJ) or neuralgia-inducing cavitational osteonecrosis (NICO). While CSR represents a radiological and morphological observation, FDOJ and NICO constitute pathological diagnoses that require histopathological confirmation. Radiological imaging alone is insufficient to establish these diagnoses. Consequently, equating CSR with FDOJ or NICO based solely on imaging findings is neither scientifically nor clinically justified.

### 3.4. Therapy

Covered Socket Residuum (CSR) may remain a purely radiological and morphological observation without an inherent clinical need for therapeutic intervention. In this context, greater emphasis should be placed on the application of additional treatment and regenerative techniques in oral and maxillofacial surgery aimed at preventing the formation of CSR, particularly by supporting bone regeneration and preventing socket collapse.

In contrast, jawbone cavitations described as FDOJ or NICO have been discussed as pathological entities and, in selected cases, may be associated with clinical symptoms requiring therapeutic management. Current treatment concepts described in the literature predominantly involve surgical decortication or curettage of the affected jawbone area with the aim of removing chronically altered or inflamed tissue. However, indications for such interventions remain controversial and should be carefully evaluated on an individual basis.

## 4. Discussion

This narrative review aimed to summarize the current understanding of jawbone cavitations, identify relevant gaps in the existing literature, and propose a unified and descriptive terminology to facilitate future research and clinical communication. Our research findings outlines very limited evidence about the etiology, understanding and therapy of jaw cavities.

### 4.1. Learning from Socket Healing

CSR has been predominantly observed within former tooth extraction sockets [10,22]. In this context, it is essential to critically re-examine the current understanding of this process. Socket healing represents a unique and complex physiological regenerative process, as it involves the simultaneous repair of both hard tissue (alveolar bone) and soft tissue (gingiva) defects [25,26,27].

Socket healing has been investigated using various experimental and clinical models [28,29,30,31,32,33]. Much of the foundational knowledge is derived from animal studies, which predominantly relied on histological analyses to characterize the healing cascade following tooth extraction [34]. Based on these observations, the classical concept of socket healing has been described as analogous to fracture healing [35]. Following tooth extraction, the socket is initially filled with a stable blood clot, corresponding to the hemostatic phase of fracture healing. This clot is subsequently reorganized into immature bone tissue, analogous to the ossification phase, and later remodeled into mature lamellar bone during the remodeling phase [25,29,34,36,37].

However, this classical model is largely based on histological observations under experimental conditions. Additionally, in clinical practice, unassisted socket healing has frequently been associated with bone atrophy and volumetric changes in the alveolar ridge [38,39]. These dimensional alterations have been explained by several complementary theories, including the critical role of the vestibular (buccal) lamella [40], the number and stability of remaining socket walls, and defect classifications based on socket morphology [38]. Furthermore, alveolar bone volume loss has been consistently correlated with the absence of biomechanical stimulation following tooth loss, highlighting the functional dependency of bone maintenance [41]. In this context, alveolar bone atrophy following tooth loss is generally accepted as a physiological process [38]. These observations have gradually justified the implementation of socket stabilization strategies [42,43], including the use of bone substitute materials for socket or ridge preservation [44]. However, despite their widespread clinical application, there remains limited evidence regarding the etiology and biological mechanisms underlying post-extraction volumetric bone atrophy.

In a recent study, we investigated the unassisted socket healing process over time using three-dimensional radiological assessment and visualization techniques. The results demonstrated a collapse of the extraction socket characterized by an inward movement of the socket walls, which was more pronounced on the buccal side than on the oral side [10]. Based on these observations, we postulated that alveolar bone atrophy may, at least in part, be related to this centripetal collapse of the socket walls.

**Figure 3:**
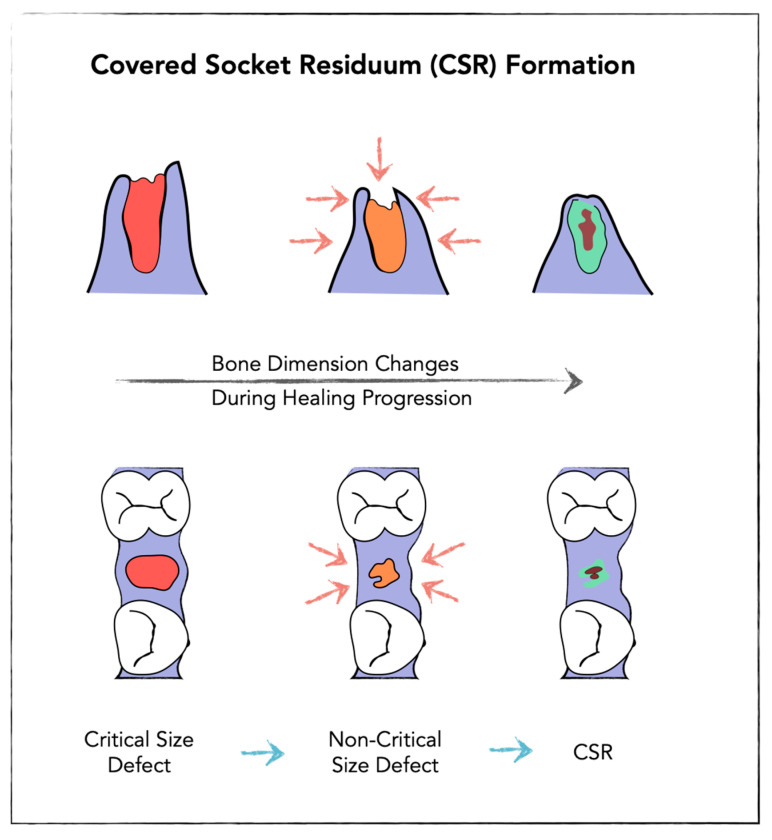
Schematic representation of post-extraction socket healing showing transformation from a Critical Size Defect → Non-Critical Size Defect → Covered Socket Residuum (CSR) formation (upper panel mesial to distal view, lower panel occlusal view).

During this process, it is conceivable that the body attempts to reduce the extraction socket from a so-called critical-size defect to a non-critical-size defect by approximation of the buccal and oral walls. As a consequence, the crestal portion of the socket may undergo more rapid ossification through appositional bone formation compared with the underlying regions [10]. This healing pattern may ultimately result in the formation of Covered Socket Residuum (CSR), characterized by a non-mineralized intraosseous area in the apical part of the socket that is covered by a mineralized crestal bone layer [22]. Basen on these observations, Thereby, this structural configuration defines the CSR as a healed but incompletely ossified socket and may present a physiological condition (Figure 3). Ongoing studies will further elucidate this mechanism and provide more information about CSR formation and its relation to the atrophic jaw.

### 4.2. Clinical Implications

The detection of CSR within an extraction socket most probably represents a physiological condition following tooth loss. Nevertheless, its identification may be of considerable clinical importance for subsequent treatment planning. Dental implants have been established as a reliable and widely accepted cornerstone of modern dentistry for many years. As dental implants are commonly placed in former extraction sites, implant placement through or adjacent to a CSR potentially compromise primary stability and may increase the risk of micromovement, fibrous encapsulation, early implant loss, or peri-implant disease, particularly when internal non-mineralized areas are not identified preoperatively. However, these hypotheses require further well-designed clinical studies to evaluate the relevance of CSR as a potential risk factor for implant-related complications and to validate the concepts proposed in this review.

Based on the data presented in this review, including evidence for the existence of jawbone cavitations—particularly CSR—the use of three-dimensional radiological diagnostics, such as cone-beam computed tomography (CBCT) [45], during dental implant planning is recommended. Such assessment enables the identification of potential CSR following tooth loss and facilitates appropriate treatment planning aimed at achieving a sufficiently mineralized alveolar bone for implant placement, thereby potentially reducing the risk of implant-related complications. In addition, the development and application of preventive strategies to minimize CSR formation may be beneficial in supporting complete bone ossification prior to implant placement.

Additionally, CSR may have a potential role in association with specific systemic conditions, as jawbone cavitations have been reported to correlate with increased systemic inflammatory activity, particularly the overexpression of RANTES/CCL5. These observations are primarily derived from associative studies, and further well-designed clinical investigations with a high level of evidence are required to clarify their clinical relevance and underlying biological mechanisms.

Overall, it is important to clearly distinguish CSR as a physiological condition resulting from tooth loss and socket healing from previously described entities such as neuralgia-inducing cavitational osteonecrosis (NICO) and fatty degenerative osteolysis of the jawbone (FDOJ), which represent pathological diagnoses and are more frequently associated with clinical symptoms.

### 4.3. Prevention and Potential Therapeutic Concepts

Considering current techniques in modern oral and maxillofacial surgery and the data presented regarding CSR, there is a clear rationale for the development of standardized and biologically sound surgical approaches following tooth extraction to support bone regeneration and minimize the formation and persistence of CSR.

Preventive strategies should primarily include atraumatic extraction techniques [46] and thorough debridement of the extraction socket to remove residual inflammatory tissue associated with the extracted tooth. This approach helps prepare the socket to support adequate bone regeneration. In addition, socket preservation measures and biologically guided augmentation protocols may be considered, depending on the specific clinical situation.

In this context, the recently described Guided Open Wound Healing (GOWH^TM^) concept may represent a clinically appropriate preventive approach to reduce the risk of CSR formation [47,48]. This concept is based on respecting the physiological remodeling processes and dynamic healing patterns of the extraction socket while preserving jaw anatomy and minimizing socket collapse.

GOWH^TM^ supports the use of bone substitute materials within the alveolar socket and soft tissue substitute materials to address the associated soft tissue defect. The primary aim of this approach is to reduce socket collapse and soft tissue scar formation, preserve alveolar dimensions, and support bone regeneration by maintaining an open but protected wound environment that promotes structured mineralization while avoiding the formation of enclosed residual intraosseous non-mineralization. Ongoing efforts are focused on establishing this treatment concept and providing comprehensive education and training for oral and maxillofacial surgeons [47].

In selected cases where elimination of CSR is considered clinically indicated—such as during implant planning or in the presence of unclear radiological findings—a thorough debridement of the affected region, followed by histopathological analysis of the removed tissue, should be performed. This approach allows a reliable distinction between physiological healing patterns and true pathological alterations. Such an evidence-based and biologically oriented strategy supports diagnostic clarity, improves reproducibility, and facilitates resource-efficient clinical decision-making.

Following CSR debridement, the application of GOWH^TM^ is proposed as a supportive strategy to promote bone regeneration by preventing wound collapse and reducing the risk of CSR recurrence.

## 5. Conclusions

Jawbone cavitations have been described for several decades; however, their biological nature and clinical relevance remain incompletely understood. This narrative review summarizes the current state of evidence, identifies key research gaps, and proposes a unified and descriptive terminology. In this context, Covered Socket Residuum (CSR) is introduced as a physiological outcome of socket collapse and incomplete ossification following tooth extraction and is clearly distinguished from pathological entities such as neuralgia-inducing cavitational osteonecrosis (NICO) and fatty degenerative osteolysis of the jawbone (FDOJ).

Recognition of CSR using three-dimensional radiological assessment, particularly cone-beam computed tomography, is clinically relevant in implant dentistry, as unrecognized intraosseous non-mineralized areas may compromise primary stability and increase the risk of implant-related complications. Accordingly, preventive and biologically guided post-extraction management strategies, including approaches such as Guided Open Wound Healing (GOWH), should be considered to support complete bone regeneration.

Overall, the available evidence regarding the etiology, prevention, and treatment of jawbone cavitations remains limited and is largely based on observational data. Well-designed prospective clinical studies integrating radiological, histological, and implant outcome parameters are required to validate the CSR concept and determine its relevance for long-term implant success.

## 6. Outlook

The present review highlights critical research gaps regarding the biological nature, etiology, and clinical relevance of jawbone cavitations, particularly Covered Socket Residuum (CSR). Future research should focus on the validation of preventive strategies aimed at minimizing CSR formation, the systematic evaluation of CSR as a potential risk factor in dental implant therapy, and the elucidation of the underlying biological and healing mechanisms associated with incomplete socket ossification. In addition, the possible relationship between CSR and systemic inflammatory conditions warrants further investigation.

To address these questions, well-designed prospective and randomized controlled clinical trials, integrating radiological, histological, and implant-related outcome measures, are essential to establish evidence-based diagnostic and therapeutic recommendations.

## Data Availability

The original contributions presented in this study are included in the article. Further inquiries can be directed to the corresponding author.
